# Failure of response to combination therapy of adalimumab and infliximab in a recalcitrant patient of severe psoriasis and major thalassemia: A case report

**DOI:** 10.1002/ccr3.2696

**Published:** 2020-02-03

**Authors:** Mohamad Goldust, Ghasem Rahmatpour Rokni, Mrinal Gupta, Torello Lotti, Marzieh Bathaei

**Affiliations:** ^1^ Mazandaran University of Medical Sciences Sari Iran; ^2^ University of Rome Guglielmo Marconi Rome Italy; ^3^ Department of Dermatology University Medical Center Mainz Mainz Germany; ^4^ Department of Dermatology University Hospital Basel Basel Switzerland; ^5^ Department of Dermatology Mazandaran University of Medical Sciences Sari Iran; ^6^ Treatwell Skin Centre Jammu India; ^7^ Department of Dermatology University of Studies Guglielmo Marconi Rome Italy

**Keywords:** adalimumab, infliximab, major thalassemia, psoriasis

## Abstract

This is the first case report of combination therapy in psoriasis with underlying thalassemia that failed after months. The relation of thalassemia with severity of psoriasis and response to treatment should be evaluated in more studies.

## Graphical abstract

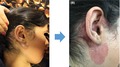

## 1 INTRODUCTION

Psoriasis is a chronic inflammatory skin disease, characterized by scaly, erythematous, well‐defined papules and plaques, with a relapsing and remitting course.^1^ A number of biologics have been approved and used in the management of psoriasis which target specific steps in the immune cascade, thereby inhibiting the immune process leading to psoriasis. Biologic therapy has significantly advanced the management of psoriasis, making complete plaque clearance an achievable goal even in patients with severe psoriasis. Tumor necrosis factor‐α is a key mediator in the pathogenesis of psoriasis. Infliximab and adalimumab, the most commonly used biologics in management of psoriasis, are TNF‐α inhibitors which have shown to reduce the disease severity and improve the quality of life.^2^, ^3^ These agents are usually used alone but severe nonresponsive cases may warrant their use together as combination therapy but many times they fail to achieve the desired results. We report a case of a 27‐year‐old woman who had history of 6 years for psoriasis with underlying major thalassemia, who was treated with a combination therapy of adalimumab and infliximab, who initially responded well to treatment but later became nonresponsive, which could be attributed to the presence of thalassemia.

## 2 CASE PRESENTATION

**Figure 1:**
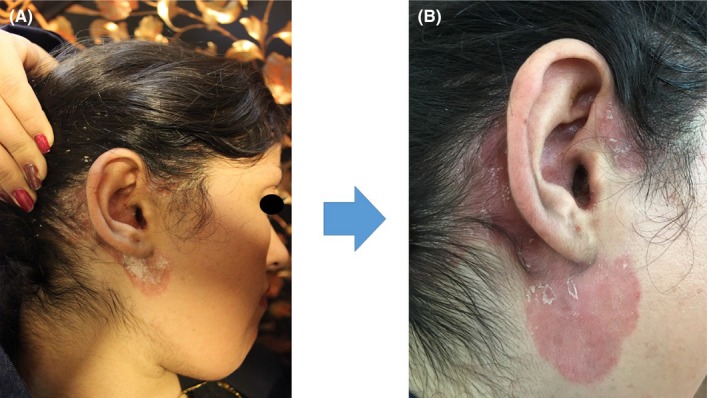
Failure of response to combination biologic therapy (A) Scalp lesion before combination biologic therapy, (B) 6 mo after treatment

A 27‐year‐old woman presented to us with a history of chronic plaque psoriasis, which initially started over the scalp with PASI of two but over the next 6 years had gradually involved the elbows and knees and the PASI became 12. She had pitting of her nails, and there was no joint involvement and features of psoriatic arthritis. Her medical history revealed that she was a case of major beta thalassemia/Hb E and she was receiving packed cell infusions every 22 days. She had iron overload related to her disease and her ferritin level was 1256 ng/mL and she was under treatment with 1 g deferoxamine. Previous systemic treatments for her included adalimumab, infliximab, and methotrexate 20 mg weekly for 2 years, which were used individually for disease control. After 2 years of treatment, the disease became nonresponsive to these drugs with gradual progression of the disease severity (Figure 1A). Her PASI was >10 with Dermatology Life Quality Index (DLQI) > 10 and body surface area (BSA) > 10%. Due to the comorbidity and the unresponsiveness to all other therapies and due to the disease progression, the case was discussed in the rheumatology board. She was started on a combination therapy of adalimumab and infliximab. On starting combination therapy with adalimumab 80 mg SC once, then, after 1 week, 40 mg SC q2wk and infliximab 5 mg/kg IV at 0, 2, and 6 weeks, then q8weeks thereafter, lesions significantly improved initially with reduction in PASI and DLQI but after 6 months the lesions started aggravating and new lesions also started appearing, unexpectedly (Figure 1B). No side effects appeared. PASI score of 1.6 and DLQI of 1 were increased to 2.4 and 3, respectively, at 24 weeks of treatment. Currently, the patient is under treatment with intralesional suspension of 1% of triamcinolone acetonide subcutaneously, with PASI of 1.2, after the second injection. DLQI and BSA are under two as well.

## 3 DISCUSSION

A large number of treatment modalities have been used for the management of psoriasis which include NBUVB phototherapy, psoralens with ultraviolet A light, retinoids, methotrexate, cyclosporine, and biologics. Anti‐TNF‐α agents are now one of the most commonly used treatment modalities which have improved the management of psoriasis. Adalimumab is well established for the treatment of moderate‐severe chronic plaque psoriasis in adults and has more recently been approved in the European Union for use in pediatric patients with severe chronic plaque psoriasis.^4^ Infliximab, a monoclonal antibody that binds with high affinity and specificity to TNF and neutralizes its biological activity, is approved for the treatment of psoriasis and psoriatic arthritis.^5^ The biologic agents are usually used alone, but in severe, nonresponsive cases, they can used in combination to achieve better disease control.^6^ Our patient, a known case of thalassemia major, showed dramatic response to combination therapy initially with reduction and clearance of lesions and reduction of PASI score and DLQI. But after six months of combination therapy, the disease stopped responding to the therapy with worsening of symptoms and PASI and DLQI scores. Atteno et al observed that in patients with psoriatic arthritis, 8% patients had thalassemia trait. They also evaluated the efficacy of anti‐TNF‐α therapy in patients with psoriatic arthritis and thalassemia and showed that the presence of thalassemia could be a negative predictor of achieving remission during treatment with TNF‐α blockers. They observed that in patients without thalassemia, all variables of disease severity improved significantly after 6 months of therapy, while in patients with thalassemia, the same variables showed a significant decrease after 12 months.^7^ Studies have shown that there is high association between psoriatic arthritis and thalassemia which has been hypothesized to be due to a genetically determined susceptibility.^8^ The presence of hemoglobinopathies like thalassemia could be a negative predictor of achieving remission during treatment with TNF‐α blockers. Studies have also shown that there is immunologic response in the form of T‐cell reduction, C4 complement reduction, changes in CD8+/ CD4+ T‐lymphocyte ratio, presence of autoantibodies, increased levels of immunoglobulins, and circulating immune complexes which may predispose to expression of an autoimmune disease such as psoriasis and psoriatic arthritis and these immunologic responses could also alter the response of psoriasis to the different treatment modalities.^9^ Presence of thalassemia trait in patients with psoriasis could be a negative predictor of achieving remission during treatment with TNF‐α blocker regimens but present data are insufficient and further studies are needed to address this issue.

## CONFLICT OF INTEREST

None declared.

## AUTHORS' CONTRIBUTIONS

All authors have read and approved the manuscript, and ensure that this is the case. Author 1: contributed to patient follow‐up and management and final approval of the version to be published. Author 2: contributed to patient management and writing article, drafted the work, and substantively revised it. Author 3: contributed to patient follow‐up and management, drafted the work, and substantively revised it. Author 4: contributed to patient management and drafting the manuscript. Author 5: contributed to patient follow‐up and management, drafted the work, and substantively revised it.
